# Effect of Co-fed Water on a Co–Pt–Si/γ-Al_2_O_3_ Fischer–Tropsch Catalyst Modified with an Atomic Layer Deposited or Molecular Layer Deposition Overcoating

**DOI:** 10.1021/acsomega.1c06512

**Published:** 2022-02-23

**Authors:** Niko Heikkinen, Laura Keskiväli, Jasmiina Palo, Matti Reinikainen, Matti Putkonen

**Affiliations:** † VTT Technical Research Centre of Finland, P.O.Box 1000, FIN-02044 VTT, Espoo, Finland; ‡ Department of Chemistry, University of Helsinki, P.O.Box 55, FIN-00014 Helsinki, Finland

## Abstract

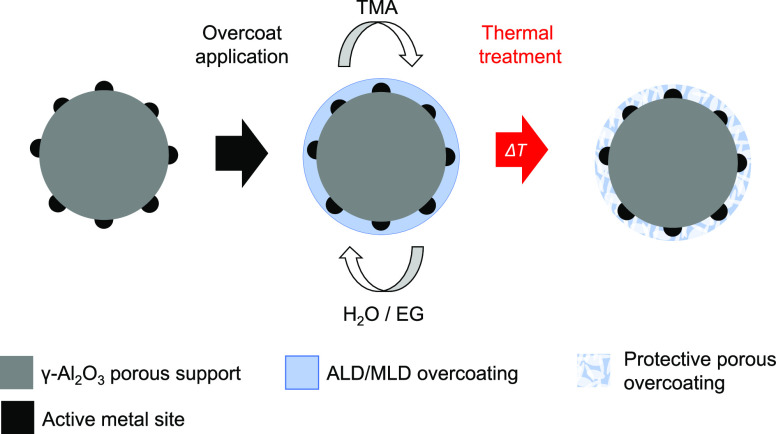

Atomic layer deposition (ALD) and molecular layer deposition (MLD) methods were used to prepare overcoatings on a cobalt-based Fischer–Tropsch catalyst. A Co–Pt–Si/γ-Al_2_O_3_ catalyst (21.4 wt % Co, 0.2 wt % Pt, and 1.6 wt % Si) prepared by incipient wetness impregnation was ALD overcoated with 30–40 cycles of trimethylaluminum (TMA) and water, followed by temperature treatment (420 °C) in an inert nitrogen atmosphere. MLD-overcoated samples with corresponding film thicknesses were prepared by using TMA and ethylene glycol, followed by temperature treatment (400 °C) in an oxidative synthetic air atmosphere. The ALD catalyst (40 deposition cycles) had a positive activity effect upon moderate water addition (*P*_H_2_O_/*P*_H_2__ = 0.42), and compared with a non-overcoated catalyst, it showed resistance to irreversible deactivation after co-fed water conditions. In addition, MLD overcoatings had a positive effect on the catalyst activity upon moderate water addition. However, compared with a non-overcoated catalyst, only the 10-cycle MLD-overcoated catalyst retained increased activity throughout high added water conditions (*P*_H_2_O_/*P*_H_2__ = 0.71). All catalyst variations exhibited irreversible deactivation under high added water conditions.

## Introduction

Fischer–Tropsch (FT) synthesis is a versatile method for converting a wide variety of feedstocks into valuable chemical products. In many cases, such as with biomass gasification or CO_2_ utilization schemes, remarkable amount of water can be present in the feed of the FT step. As the removal of water by condensing affects the overall economics of the process, it is important to understand how catalyst activity and stability can be retained with moist feeds.

In addition to water from upstream processes, water is an inherent component of cobalt-catalyzed FT synthesis. For each mole of converted CO, 1 mol of water is generated. Several studies discuss the effect of added water on the catalyst performance.^1−5^ Often reported effects include increased C_5+_ and lowered CH_4_ selectivity,^4,5^ while catalyst activity and deactivation are dependent on several factors including support composition, cobalt loading, cobalt crystallite size, promoter selection, reactor type, and reaction conditions.^4,6,7^ Bertole et al.^3,8^ have shown that water enhances catalyst activity by lowering the CO activation energy barrier and increasing the carbon species (CH_*x*_) surface coverage via an assisted CO dissociation rate on the catalyst surface. In addition to a positive effect on the activity, the higher coverage of the reactive monomer species is suggested to increase C_5+_ selectivity through an enhanced polymerization rate without increasing chain termination probability. The increased activity has mainly been reported for TiO_2_- and SiO_2_-supported catalysts,^9,10^ and decreased activity has mainly been reported for Al_2_O_3_-supported catalysts with narrow pores below 13 nm.^2,5,11^ Although catalysts supported on narrow-pore γ-Al_2_O_3_ show a lower CO conversion rate, interestingly, the C_5+_ selectivity is increased without exception.^11−13^

Bertole et al.^3^ indicated the positive effect on the FT reaction rate of moderate water addition to unsupported Co catalysts with a large particle size. These positive effects were considered to be mainly kinetic in nature as mass transfer limitations on unsupported Co catalysts were assumed to be negligible. Although positive results were gained with moderate water amounts, increasing water partial pressure resulted in lowered activity. According to Rytter et al.,^14^ this lowered activity could result from suppressed hydrogenation reactions on the catalyst active sites upon high water partial pressure. In addition to activity effects, an added water amount has been shown to have reversible and irreversible effects on the catalyst performance. Fratalocchi et al.^4^ studied co-fed water on the Co/γ-Al_2_O_3_ catalyst in a fixed-bed tubular reactor and reported both reversible and irreversible effects on the FT reaction rate. They showed that operating at a low partial pressure regime (*P*_H_2_O_/*P*_H_2__ < 0.32) had no remarkable effect on the FT reaction rate, although slow deactivation induced by co-fed water was observed. Dalai and Davis^15^ reported that water-induced irreversible deactivation with alumina- and titania-supported catalysts was only found with a high partial-pressure regime (*P*_H_2_O_/*P*_H_2__ > 0.6). Similar findings were reported by Borg et al.^11^ with a Co–Re/γ-Al_2_O_3_ (20/0.5 wt %) catalyst, where a moderate level (*P*_H_2_O_/*P*_H_2__ = 0.4) indicated the positive effect of the FT rate on the average pore diameter (20.8 nm) and a negative rate effect on samples with small pores (11.6 nm). In addition to pore diameter dependency, they concluded that increased water partial pressure (*P*_H_2_O_/*P*_H_2__ = 0.7) at the reactor inlet resulted in a decreased FT rate as well as irreversible deactivation.

Deactivation in low-temperature cobalt-based FT synthesis may occur through active site reoxidation, carbon species formation on the catalyst surface, carbidization, surface reconstruction, cobalt sintering, metal–support solid-state reactions, and mechanical attrition.^16^ In order to decrease the rate of deactivation, solution-based overcoating methods have been proposed for the prevention of the leaching of the active metal in liquid-phase reactions and particle sintering in high-temperature (>500 °C) reactions.^17−19^ However, due to the challenge of controlling the amount of the deposited material, solution-based methods often form unintentionally porous, agglomerated, and relatively thick overcoatings. Different from solution-based methods, solid-state synthesis can be used to produce overcoatings with high conformality and gain precise control over the deposited material.^20^ In particular, atomic layer deposition (ALD) and molecular layer deposition (MLD) are suitable for heterogeneous catalysis applications.^21^ Several successful examples of ALD metal oxide overcoatings such as Al_2_O_3_, TiO_2_, and SiO_2_ can be found in the related literature.^22−30^ Seo et al.^29^ used a TiO_2_ overcoating on a Ni catalyst powder for a CH_4_ dry-reforming reaction. The overcoating was found to increase the activity of the Ni-reforming catalyst and reduce carbon deposit formation. Similarly, Lu et al.^30^ reported that an 8 nm thick (45 cycles) alumina overcoating prevented particle sintering and eliminated carbon formation almost completely in a Pd/Al_2_O_3_-catalyzed ethane dehydrogenation reaction at 650 °C. Their approach was to calcine (700 °C) an overcoated catalyst prior to the reaction, enabling gaseous reactants access to the active sites through the formed porous ALD overcoating. They argued that the alumina overcoat could selectively block low-coordinated surface sites (edge and corner atoms) that are prone to catalyst coking while leaving the planar surfaces available for the dehydrogenation reaction. In addition to gas-phase reactions, ALD overcoatings have been studied in liquid-phase reactions.^22,28,31^ Lee et al.^22^ showed that a 30-cycle overcoating can prevent cobalt leaching and sintering in a furfural aqueous-phase hydrogenation reaction. The sintering of cobalt particles was not observed when the catalyst overcoating was subjected to calcination at 600 °C before the hydrogenation reaction.

Compared to ALD overcoatings, fewer examples of protective MLD overcoatings are available. Typically in MLD, organic difunctional molecules are used as precursors instead of traditional ALD oxygen sources, such as H_2_O or O_3_.^32^ MLD coatings have been used in catalytic applications, for example, in stabilizing Pt nanoparticles in the oxidation reactions of CO and using Ni in the dry reforming of methane.^33−35^ Regarding CO oxidation reactions, Liang et al.^33^ observed that a catalyst with a porous overcoating was more stable against sintering during calcination in air, but the activity of the overcoated Pt nanoparticles was lower due to the small pore size of the overcoat. In the case of methane dry reforming, Gould et al.^34^ used “ABC”-type MLD overcoatings, where A refers to trimethylaluminum (TMA), B refers to ethanolamine, and C refers to maleic anhydride. They concluded that five MLD cycles yielded the highest steady-state methanation rate compared with an uncoated catalyst and all porous overcoatings stabilized the catalyst against sintering under high-temperature reforming conditions. Ingale et al.^35^ compared alumina and alucone [TMA + ethylene glycol (EG)] overcoatings on Ni/SiO_2_ catalysts in a methane-reforming reaction. They stated that ALD-modified Ni/SiO_2_ catalysts had decreased methane dry-reforming activity, supposedly due to the formation of inactive NiAl_2_O_4_ species. The situation was different with MLD-modified Ni/SiO_2_ catalysts as they showed increased activity and stability under harsh methane-reforming conditions.

A common factor in these ALD/MLD overcoatings is the necessity for a pretreatment before the reaction. ALD-overcoated catalyst treatment is mainly conducted in an inert atmosphere with elevated temperatures (400–700 °C), whereas MLD overcoatings are oxidized in air or etched with water vapor. The purpose of treatment is to create porosities and reopen catalyst active sites for the reaction.^22,30,31^ Interestingly, after the formation of the porous overcoating, positive effects have been reported on catalyst activity, selectivity, and/or deactivation resistance. Positive effects have been suggested to result from coking prevention, leaching, and sintering resistance^22,30^ as well as from the modified reaction environment on the catalyst surface.^29^

This paper addresses the effect of ALD and MLD overcoatings on FT catalysts. Both ALD and MLD overcoatings were applied on the same Co–Pt–Si/γ-Al_2_O_3_ catalyst and subjected to relevant FT reaction conditions and co-fed water. To the authors’ knowledge, this is the first study on the effect of added water on FT catalysts overcoated with ALD and MLD.

## Results and Discussion

### Effect of an Overcoat on the Deactivation Rate

**Figure 1:**
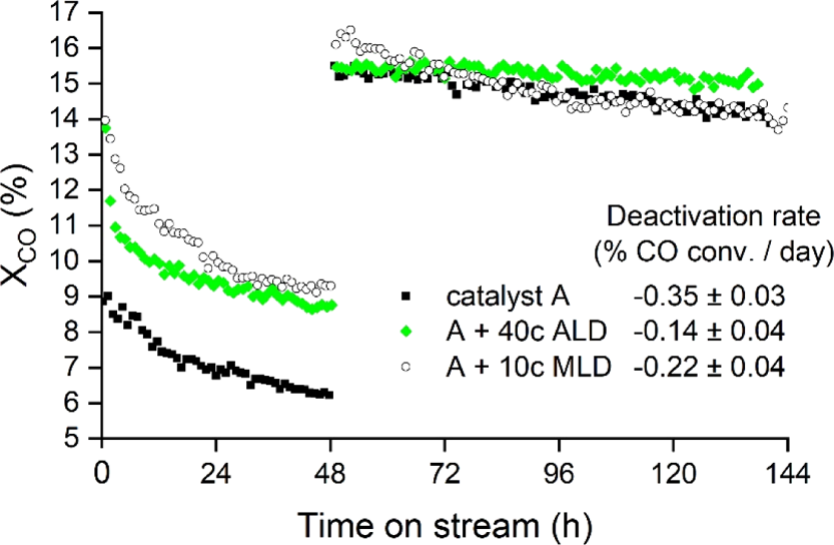
Catalyst deactivation rate, calculated from the dry run period (48–144 h). After 48 h, CO conversion is adjusted to 15% with feed gas flow reduction.

**Figure 2:**
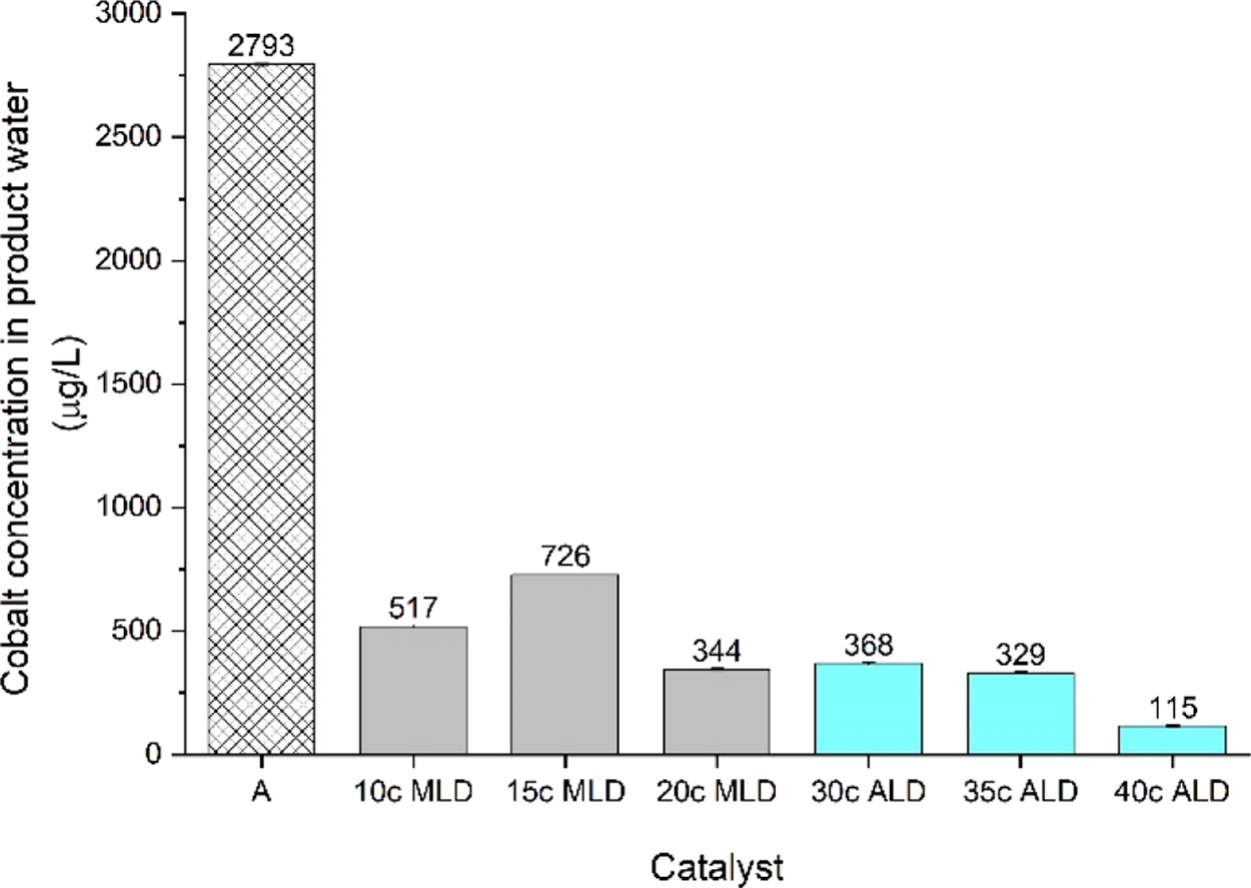
ICP–MS analysis results from water samples collected after step B.

Two experiments were conducted to address the overcoat effect on the deactivation rate. These experiments were a dry run reaction experiment (see Figure 1) and an inductively coupled plasma–mass spectrometry (ICP–MS) cobalt analysis on collected water samples (see Figure 2). Figure 1 presents the dry run experiment without added water, where the initial activity phase endured from 0 to 48 h. After the initial activity phase, the reaction flow was adjusted to achieve a similar conversion level with all catalyst samples. The dry run deactivation rates were determined from reaction period 48 to 144 h for non-overcoated, 10c MLD, and 40c ALD catalyst samples as the CO conversion percentage loss per 24 h. During the examined period [48–144 h time-on-stream (TOS)], the 40c ALD catalyst showed a clear indication of slower deactivation, with the deactivation rate decreasing from that of the non-overcoated catalyst (−0.35 to –0.14% CO conversion/day).

**Figure 3:**
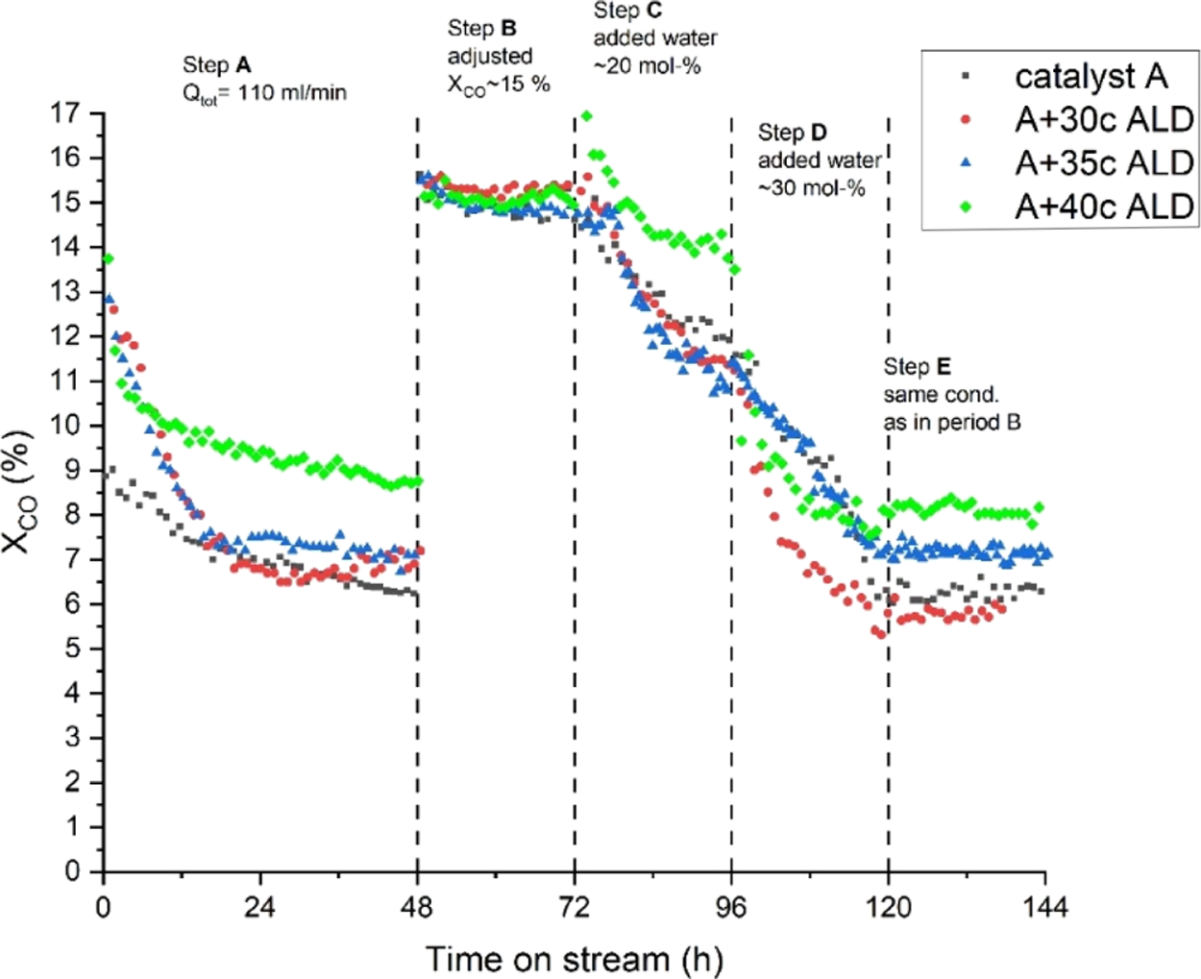
Overall ALD catalyst activity during the reaction steps.

**Figure 4:**
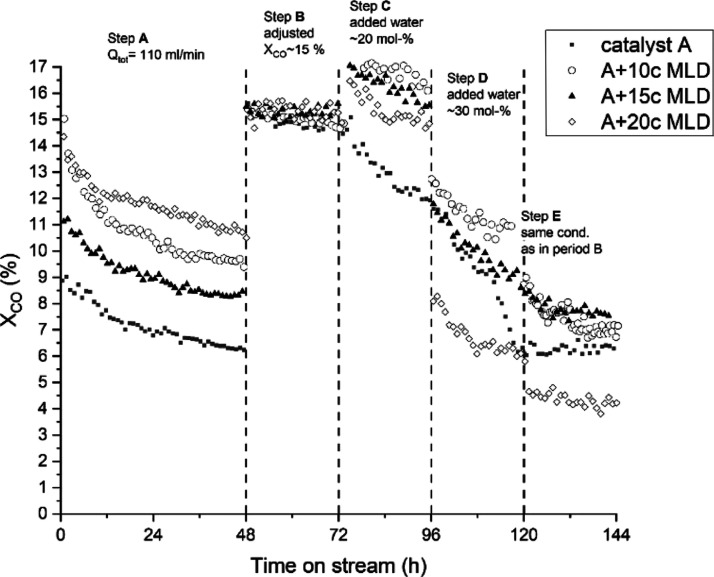
Overall MLD catalyst activity during the reaction steps.

In addition to the dry run FT reaction experiment presented in Figure 1, ICP–MS cobalt analysis was conducted on collected water samples. The water samples were analyzed for cobalt content from a cold trap at the end of step B (see Figures 3 and 4, TOS 72 h, wet run experiments). Figure 2 presents the ICP–MS results, where the non-overcoated catalyst water sample contained 2793 μg/L cobalt. Overcoated catalysts showed cobalt concentrations between 115 and 726 μg/L. The inconsistency from Figure 2 related to the 15c MLD catalyst was also observed in the reaction experiments (see Figure 4). According to Dameron et al.,^36^ this inconsistency results from the unpredictable growth of the MLD alucone (EG + TMA) overcoating with less than 100 MLD cycles. The ICP–MS results in Figure 2 indicate that a higher ALD film density and cobalt active sites provide protection against added water conditions. The ALD overcoating’s higher density was apparent from the N_2_ sorption measurements (Table 1), where the Brunauer–Emmett–Teller (BET) surface area decreased upon thickening the ALD overcoat.

**Table 1:** Nitrogen Adsorption/Desorption Measurement Results for the BET Surface Area, Pore Volume, and Pore Size^a^

catalyst	BET surface area (m^2^/g)	pore volume (mL/g)	pore size (nm)
support (Puralox SCCa 5/150)	140/-	0.46/-	13.2/-
catalyst A	105/-	0.26/-	9.3/-
A + 10c MLD	-/108	-/0.28	-/9.8
A + 15c MLD	-/108	-/0.28	-/9.7
A + 20c MLD	-/100	-/0.24	-/8.8
A + 30c ALD	21/81	0.05/0.16	6.2/8.4
A + 35c ALD	16/75	0.05/0.14	5.8/8.0
A + 40c ALD	8/60	0.04/0.11	5.7/7.9

a Results are presented for MLD and ALD catalyst samples. The values are given for before/after thermal treatment.

### Effect of Added Water on Catalyst Activity

The effect of water addition on the catalyst activity is presented in terms of CO conversion as a function of TOS. Depending on the ALD-overcoated catalyst activity in step A under a fixed reaction flow, catalyst CO conversion was stabilized between 7 and 9%. In order to compare catalyst activity and selectivity during added water conditions, it was important to set all catalysts in the same conversion range in step B.

The first water addition (Figure 3, step C) had a negative effect on all catalysts. However, the effect was less severe for the 40c ALD catalyst with ∼1% unit decrease in CO conversion, whereas the other catalysts showed a decrease of ∼3% units. This decreasing activity response to water addition is typical for inert and narrow pore γ-alumina.^1,2,10,11,37,38^ Decreased activity could follow from a combination of several deactivation mechanisms. With non-overcoated catalyst A, the decreased FT reaction rate has been associated with the oxidation of highly dispersed cobalt particles, the oxidation of cobalt surface layers, and water-induced active metal sintering.^39^ Although these deactivation mechanisms may contribute to ALD samples to some extent, the decreased activity was mainly expected to result from the formation of cobalt aluminates in added water conditions^39,40^ and porous overcoat filling with the produced and added water.

According to several studies,^1,5,11^ the effect of added water depends on the catalyst pore size. A negative effect on activity has been reported for catalysts with a narrow pore size (<13 nm)^2,5,12^ and a positive effect for larger pore sizes. Interestingly, the 40c ALD catalyst with a pore size of 7.9 nm (see Table 1) did not lose its activity significantly when a moderate amount (20 mol %) of water was added. However, the high co-fed water addition period (step D) led to an instant decrease in activity, which could not solely relate to catalyst deactivation. Instead, this could be an indication of the porous ALD overcoat filling with condensed water and/or the oversaturation of the cobalt surface by water molecules, both decreasing the amount of active surface carbon.^1,3^ Interestingly, at TOS 108 h, the 40c ALD catalyst activity decrease seems to stabilize after the presumed pore filling and remains at the same level as step E.

Figure 4 presents the overall MLD catalyst activities during the experiments. At the initial activity phase (step A) with a fixed reaction flow of 110 N mL/min, all MLD catalysts had increased activity compared with non-overcoated catalyst A. CO conversion at the end of step A was 11.0, 9.5, 9.0, and 6.5% for the 20c, 10c, and 15c MLD and non-overcoated catalysts, respectively. Interestingly, the 15c MLD catalyst showed the lowest activity among the MLD-treated catalysts during step A. A similar result was present in ICP–MS measurements shown in Figure 2. This finding supports the suggestion of Dameron et al.^36^ as they presented in their study unpredictable growth of the MLD alucone coating with EG and TMA. Due to the chemical structure of EG, the molecule can react either with one or two −AlCH_3_ species on the growing alucone film. Since EG molecules can react twice with the −CH_3_ surface species, further growth can be prevented on some parts of the film.^36^ For this reason, the growth of the MLD coating is probably uneven and variation in thicknesses might appear with a low number of overcoating cycles (<100 cycles).

**Figure 5:**
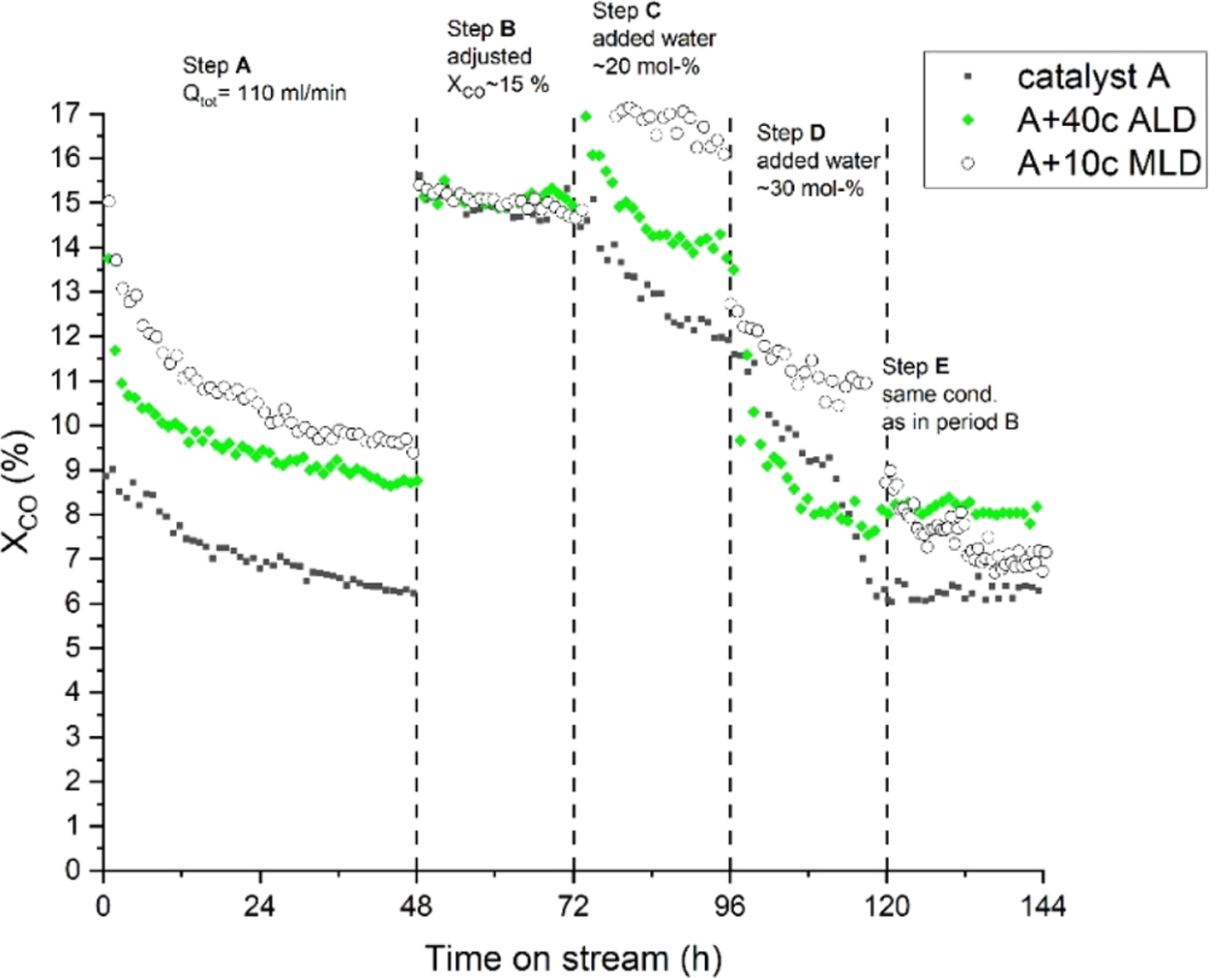
Overall catalyst activity comparison between catalyst A, 10c MLD, and 40c ALD.

In Figure 4, step C, upon first water addition, MLD catalysts showed better activity compared with that of the uncoated catalyst A. Although the 10c MLD catalyst had increased activity during added water periods, at step E (see Figure 5), the CO conversion stabilized to a lower value compared with that of the 40c ALD catalyst. This could be an indication of the structural instability of the MLD overcoating in high added water conditions, whereas the ALD catalyst, having a denser overcoating, showed less activity degradation at step E. The instability of the MLD overcoating during added water conditions could be also seen in the 20c MLD catalyst. Although having a high initial activity and a positive effect from moderate added water (as seen in Figure 4), the activity of the 20c MLD catalyst degraded under high added water conditions in step D and severe irreversible activity loss took place when the catalyst returned to dry conditions at step E.

### Catalyst Characterization

Nitrogen sorption measurement results are presented in Table 1. The surface area, pore volume, and pore size of the catalysts modified with MLD remained very similar to catalyst A, whereas ALD-overcoated catalysts show a notable decrease, especially in surface area and pore volume. The difference between MLD and ALD catalysts was assumed to result from the different methods applied for creating the porosity of the overcoat (see Figure 9). ALD-overcoating porosity was created from the deposited Al_2_O_3_ with an inert atmosphere temperature ramp, resulting in amorphous film cracking, thermal expansion, and structural changes due to dehydration^41^ and residual carbon removal.^30,42^ With TMA + EG-derived MLD films, the porosity was created upon organic precursor removal in an oxidative atmosphere, leaving a more porous Al_2_O_3_ layer.^36,43,44^ Furthermore, it has been studied that the heating rate has an effect on the film thickness, porosity, and pore shape in MLD materials.^44^ Van de Kerckhove et al.^44^ deposited alucones (TMA + EG) and discovered that a slower ramp rate results in higher porosity. By applying their findings to this study, the total estimated MLD film thickness decrease was 47% and the formed porosity was 25% (*V*_pore_/*V*_film_). The estimated film thickness decrease for ALD overcoatings was <5%.^45^ The formed porosity was not estimated for ALD overcoatings.

**Figure 6:**
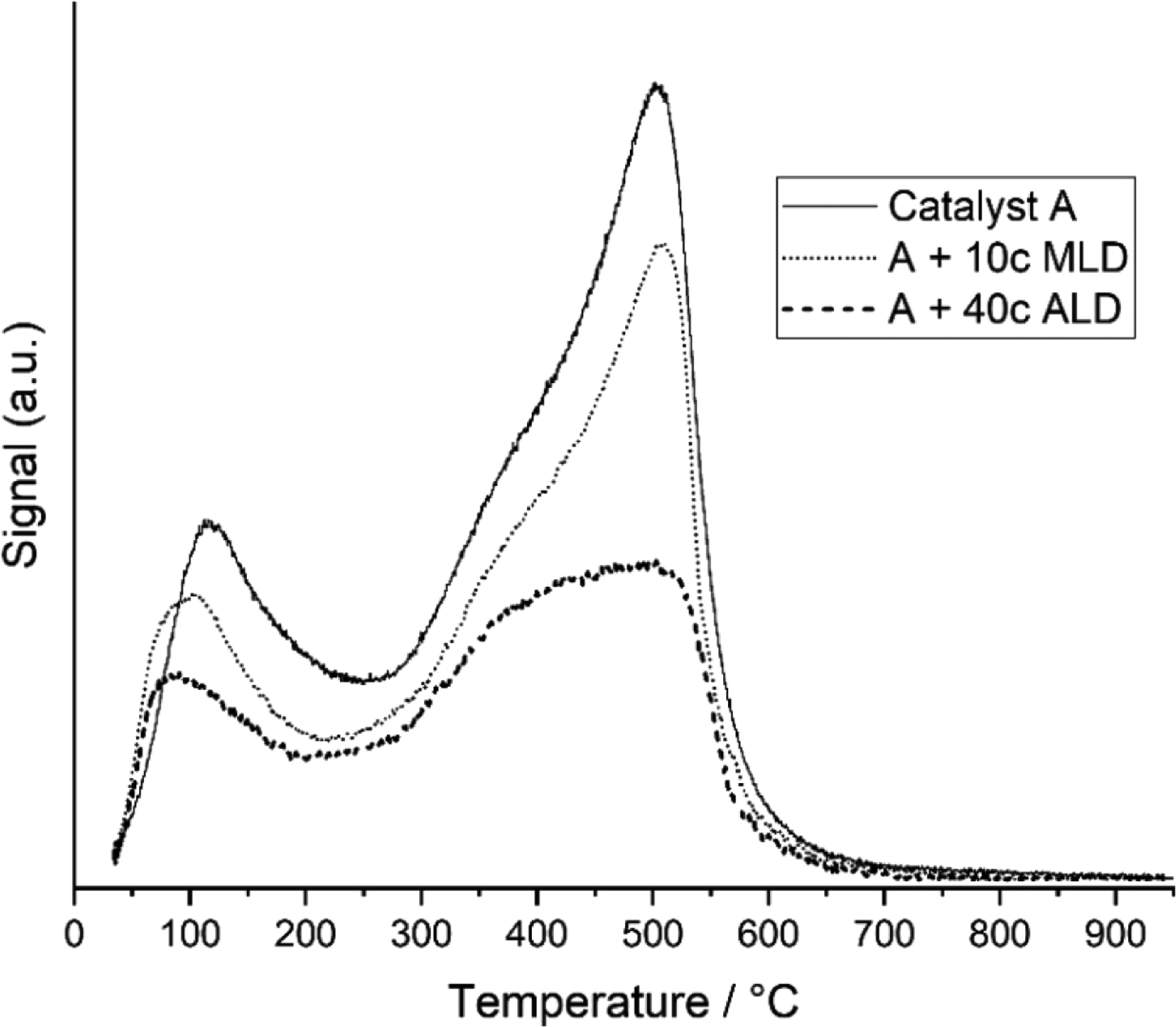
CO-TPD spectra of 10c MLD, 40c ALD, and non-overcoated catalyst A.

Temperature-programmed desorption (TPD) is a useful tool to reveal information about the CO–metal bond strength and active site types.^46,47^Figure 6 presents the CO-TPD spectra for 10c MLD, 40c ALD, and non-overcoated catalyst A. Three peaks were identified with the mass spectrometer as CO desorption peaks at temperature 100, 400, and 500 °C. These CO peaks in Figure 6 were assigned as weakly bound CO (100 °C) and tightly bound CO (400 and 500 °C) on catalyst active sites.

The results in Table 2 show that although the initial CO coverage is reduced with overcoated catalysts, strong CO binding sites are promoted with overcoated catalysts. Fu and Bartholomew^48^ have shown that CO adsorbs strongly on sites that are active for CO hydrogenation, resulting in an increased CO dissociation tendency. This could explain the enhanced overall activities for overcoated catalysts, where the tightly bound CO fraction increased from 72 to 74% and 86% for non-overcoated catalyst A, 10c MLD, and 40c ALD, respectively. This was assumed to result from modified active sites being susceptible to sub-carbonyl and bridged CO adsorption.

**Table 2:** CO-TPD Results

catalyst	pulsed CO uptake (μmol/g_cat_)	weakly bound CO (%)	tightly bound CO (%)
catalyst A	81.2	28	72
A + 10c MLD	70.4	26	74
A + 40c ALD	56.4	14	86

H_2_-temperature-programmed reduction (TPR) and H_2_ chemisorption were used to extract information from the available cobalt sites. Table 3 summarizes H_2_ chemisorption and H_2_-TPR results and estimations for cobalt dispersion and cobalt particle size. As cobalt particles were partly covered by the overcoating, dispersion estimation was not reasonable without H_2_-TPR measurement. The extent of reduction (EOR) and dispersion shown in Table 3 are only reported for overcoated catalysts with the best reaction performance (see Figure 5).

**Table 3:** Cobalt Dispersion, Particle Size, and the EOR Results from Hydrogen Chemisorption and TPR Measurements

catalyst	H_2_ uptake (μmol/g_cat_)	cobalt EOR at 400 °C (%)	acobalt dispersion (%)	bcobalt particle size (nm)
catalyst A	55.1	87	3.6	27
A + 30c ALD	49.4			
A + 35c ALD	46.7			
A + 40c ALD	43.3	86	2.8	
A + 10c MLD	57.2	87	3.7	
A + 15c MLD	54.3			
A + 20c MLD	51.5			

a Equation 1.

b Equation 2.

**Figure 7:**
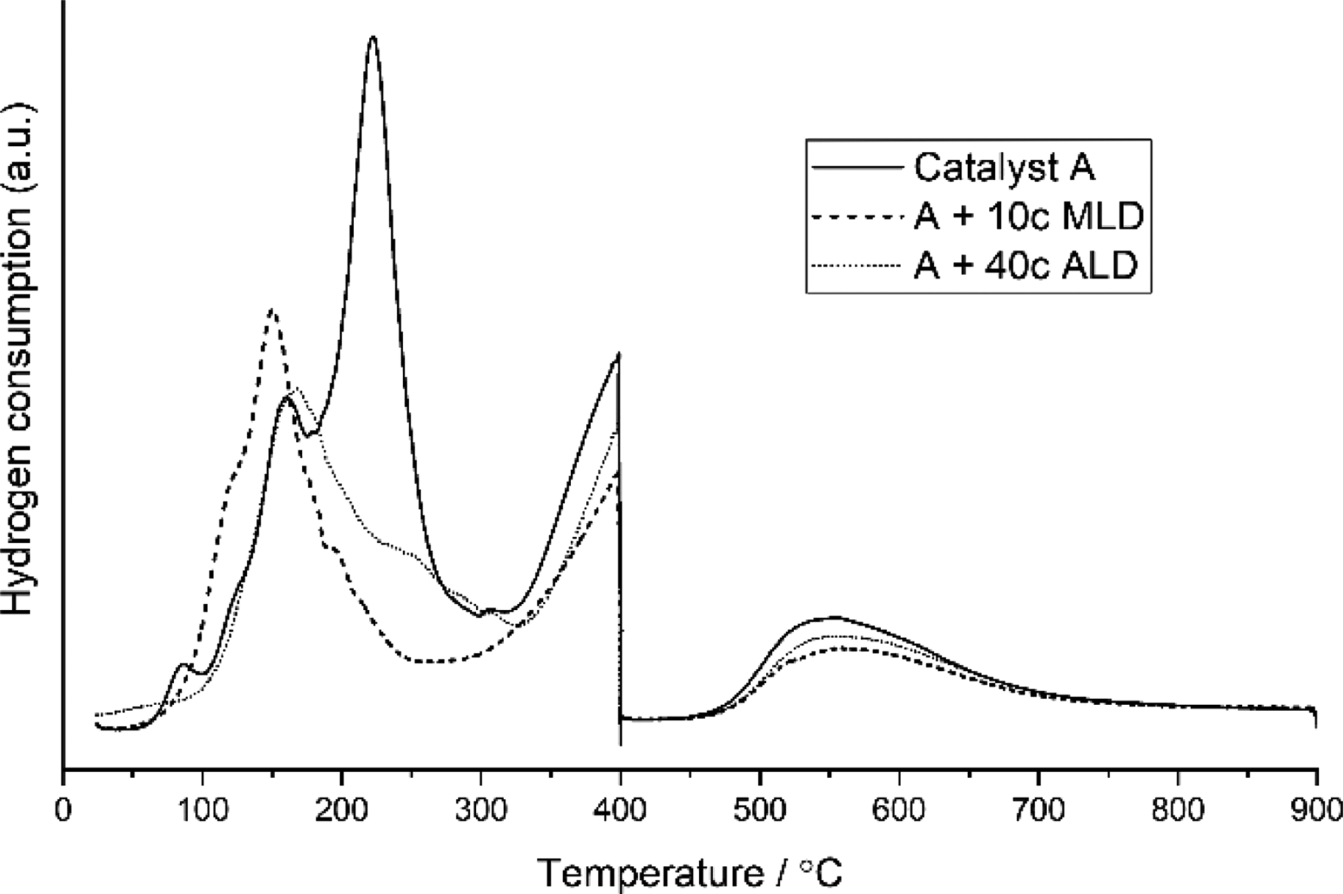
TPR curves for non-overcoated and overcoated catalysts.

The EOR shown in Table 3 was determined from the TPR curves in Figure 7. The first shoulder peak of the “Catalyst A” curve at 150 °C was assumed to result from nitrate residue removal^49,50^ and the first main peak at 225 °C from Co_3_O_4_ reduction to CoO. The second main peak at 400 °C was associated with the reduction of CoO to metallic Co. Peak tailing after 600 °C was the reduction of cobalt–support complexes.^49^ The estimated EOR values are similar among all measured samples. However, it was apparent that hydrogen during TPR only interacted with the available cobalt sites, excluding cobalt metal sites covered by ALD or MLD overcoating. This was indicated by H_2_-TPR curves in Figure 7, where overcoated catalyst hydrogen consumption was decreased compared to that of the non-overcoated catalyst during the step-wise reduction process.

### Effect of Added Water on Catalyst Selectivity

**Figure 8:**
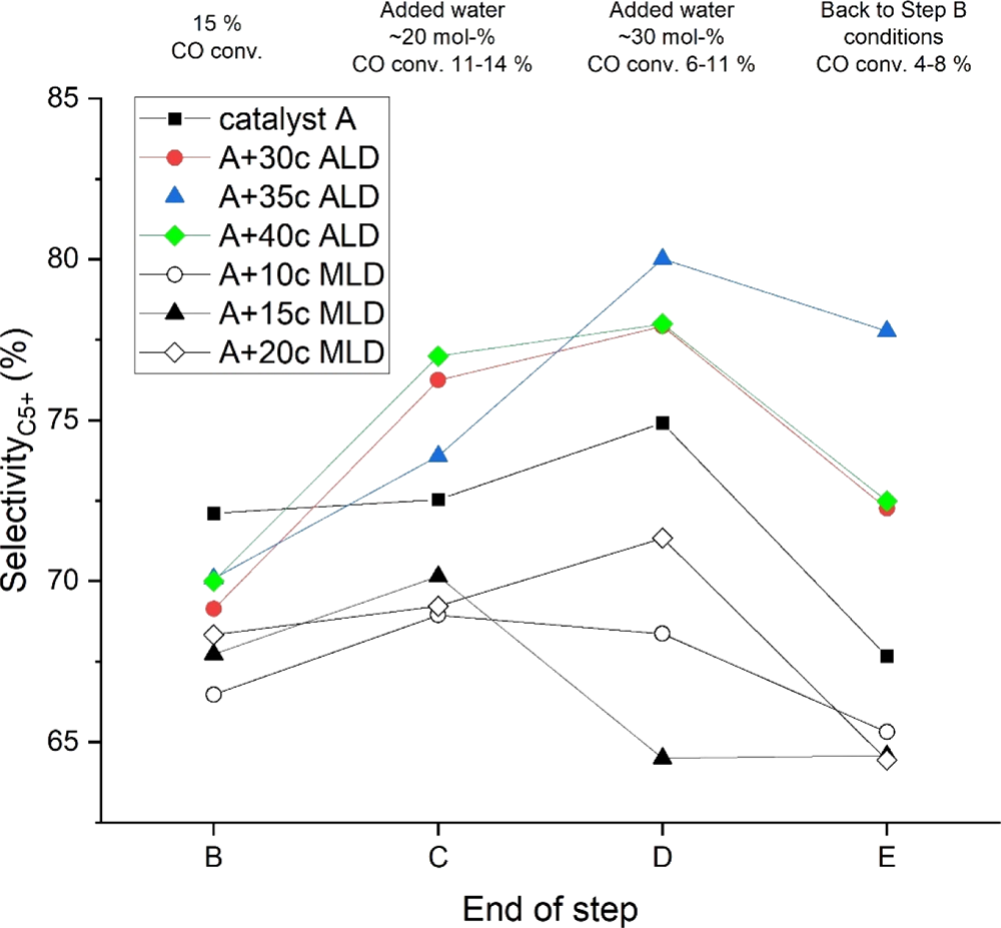
C_5+_ hydrocarbon selectivity at the end of each experimental step.

Water has been shown to increase the chain propagation α-value, resulting in enhanced C_5+_ selectivity.^4,5^ The results shown in Figure 8 support this observation as all the catalysts showed increased C_5+_ selectivity during moderate water addition (*P*_H_2_O_/*P*_H_2__ = 0.42). However, upon increased water partial pressure at the reactor inlet (*P*_H_2_O_/*P*_H_2__ = 0.71), 10c and 15c MLD catalysts showed a decreasing C_5+_ trend compared with other catalysts. This was assumed to result from water filling the MLD overcoating pores, suppressing hydrogenation reactions. At the same time, the 20c MLD catalyst showed the opposite C_5+_ selectivity trend in step D. This was assumed to result from 20c MLD overcoating degradation during step D conditions (*P*_H_2_O_/*P*_H_2__ = 0.71), resulting in more open active sites for the FT reaction. Due to the overcoating degradation, the 20c MLD C_5+_ trend remained similar to that of the non-overcoated catalyst. Table 4 gives product selectivities at a comparable conversion level (end of step B), wax fraction (C_40_–C_60_) Anderson–Schulz–Flory (ASF) α-values, ASF α-values for step B and D gaseous fraction (C_4_–C_6_), and step D methane selectivity. During step B, methane selectivity increased with all overcoated samples. Gas fraction α-values corresponded well with decreasing methane formation. Added water conditions resulted in increased α-value and decreasing methane selectivity. Exceptions were A + 35c ALD and A + 15c MLD catalysts. Addressing the reasons for A + 35c ALD increasing the C_4_–C_6_ α-value and methane selectivity was challenging due to different conversion levels between steps B and D. Catalyst A + 15c MLD showing an α-value decrease might be related to the unpredictable growth of the MLD alucone (EG + TMA) overcoating. Wax fraction (C_40_–C_60_) results show a decrease in all α-values for overcoated catalysts. Although ALD-overcoated samples had increased C_5+_ selectivity and C_4_–C_6_ α-value during added water conditions, these periods were short compared to the full TOS. The absence of CO_2_ (≪ 500 vol-ppm) from the reaction products indicated the water gas shift reaction to be negligible within all catalyst samples.

**Table 4:** Product Selectivity at Reaction Steps B and D^a^

sample collected	step B	step B	step B	after experiment	step B	step D	step D
catalyst	CH_4_ selectivity (m %)	C2–C4 selectivity (m %)	C4–C6 olefin to paraffin ratio	wax fraction α-value	gas fraction C_4_–C_6_ α-value	CH_4_ selectivity (%)	gas fraction C_4_–C_6_ α-value
catalyst A	12.6	14.9	0.60	0.90	0.731	11.1	0.742
A + 30c ALD	18.5	13.5	0.15	0.87	0.710	13.1	0.761
A + 35c ALD	13.3	16.7	0.49	0.89	0.712	13.9	0.753
A + 40c ALD	18.1	11.9	0.14	0.87	0.712	16.0	0.745
A + 10c MLD	17.8	15.2	2.10	0.87	0.704	12.2	0.714
A + 15c MLD	16.4	15.6	1.82	0.88	0.709	17.6	0.667
A + 20c MLD	17.4	14.1	1.69	0.87	0.710	10.5	0.706

a Anderson–Schulz–Flory α-values determined from wax samples collected after the experiment and α-values from step B and D gas fraction online analysis.

## Conclusions

A catalyst with 21.4 wt % cobalt, 0.2 wt % platinum, and 1.6 wt % silicon on a γ-Al_2_O_3_ support was prepared by incipient wetness co-impregnation and overcoated with ALD and MLD. The prepared ALD- and MLD-overcoated catalysts were thermally treated to create porosity on the deposited film. The ALD catalysts were thermally treated under an inert nitrogen flow at 420 °C. The MLD overcoatings were annealed in synthetic air at 400 °C. Synthetic air was used to oxidize the carbonaceous precursor, EG, from the deposited overcoating layer. These overcoated and annealed catalysts were studied in a tubular reactor for the Fischer–Tropsch (FT) reaction (200 °C, 20 bar, H_2_/CO ratio 2.0) and compared against a non-overcoated catalyst.

Our results support previous studies on added water partial pressure regimes, where moderate water addition (*P*_H_2_O_/*P*_H_2__ = 0.4) had a negative effect on the FT rate with γ-Al_2_O_3_-supported catalysts.^4,11^ However, the addition of MLD overcoating resulted in the opposite as the activity was increased during moderate added water conditions. Furthermore, in addition to moderate water partial pressure regime, the previous literature findings^4,6^ were confirmed for water-induced irreversible deactivation under a high partial pressure regime (*P*_H_2_O_/*P*_H_2__ > 0.6) for all the studied catalysts. Especially, the thickest MLD overcoated catalyst (20 cycles MLD) showed indications of overcoat degradation during added water periods. The ALD-overcoated catalyst behavior was different due to the denser overcoating. The ALD-overcoated catalysts did not achieve as high activity as the MLD catalysts. However, the ALD catalyst had better stability due to the decreased cobalt leaching during the FT reaction and enhanced resistance to irreversible degradation after added water conditions.

## Materials and Methods

### Catalyst Preparation

Step-wise co-impregnation was used to prepare the Co–Pt–Si/γ-Al_2_O_3_ catalyst. In the first step, a γ-Al_2_O_3_ support (Puralox SCCa 5-150, *S*_BET_ 140 m^2^/g, *V*_pore_ 0.46 N mL/g, and *d*_pore_ 13.2 nm) was dried at 100 °C under vacuum for 1 h. The support was then co-impregnated twice with an aqueous solution of cobalt nitrate [Co(NO_3_)_2_·6H_2_O] and platinum (IV) nitrate [Pt(NO_3_)_4_]. In the final step, tetraethoxysilane [Si(OC_2_H_5_)_4_] in ethanol solution was impregnated. After each impregnation step, the catalyst was dried in a rotary vacuum evaporator and calcined at 300 °C for 4 h. The resulting catalyst nominal loadings were 21.4 wt % cobalt, 0.2 wt % platinum, and 1.6 wt % silicon. In this study, “catalyst A” refers to Co–Pt–Si/γ-Al_2_O_3_ and “catalyst” samples with an overcoating are denoted as “35c ALD” or “20c MLD”, where the number represents deposition cycles.

The co-impregnated catalyst was divided into three batches. The first batch had no overcoating, the second batch was overcoated with ALD (30, 35, and 40 cycles), and the third batch was overcoated with MLD (10, 15, and 20 cycles).

Reference samples for thickness measurements were deposited by using the pulsing scheme of 1 s/80 s/2 s/80 s for TMA/purge/EG/purge. The measured growth per cycle (GPC) of 2.5 Å/cycle for the TMA + EG MLD process on reference silicon substrates was similar to that in the study of Van de Kerckhove et al.^44^ and lower than that of Dameron et al.^36^ reported at 85 °C (4.0 Å/cycle), although they noticed a rapid decrease of GPC to 0.4 Å/cycle if temperature was increased to 175 °C. The number of MLD deposition cycles was adjusted to 15 cycles for catalyst overcoating to result thicknesses comparable with those of the 35c ALD Al_2_O_3_ from TMA + H_2_O. Approximately 3.0 g of the catalyst powders with a particle size of 50–150 μm were coated by using a Picosun POCA powder coating system, where carrier gas and pulsed precursors are passed through an ultrasonically agitated catalyst bed. Since the surface area is significantly higher than that in planar substrates, long precursor pulse and purge times were required. It was observed that pulsing cycles of 1 s/80 s/2 s/80 s were sufficient to prevent precursor mixing and uneven growth.

**Figure 9:**
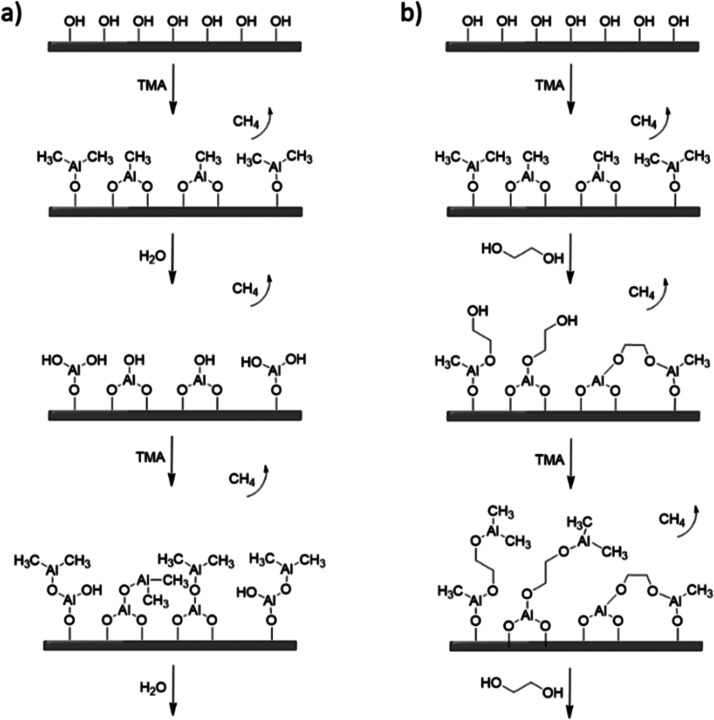
One reaction cycle and film formation for the (a) ALD process, TMA + H2O and (b) MLD process, TMA + EG.

All ALD and MLD experiments were performed with the Picosun R-200 ALD equipment. N_2_ (purity 99.999%) from liquid nitrogen was used as a carrier gas. For Al_2_O_3_ depositions at 150 °C, TMA (purity 99.999%, Sigma-Aldrich) and deionized water were used as a metal precursor and oxygen source, respectively. MLD coatings were prepared with sequential pulsing of TMA and EG (purity >99%, Sigma-Aldrich) at 90 °C. EG was evaporated at 80 °C. Figure 9 presents one deposition cycle for (a) ALD and (b) MLD. Prior to actual overcoating of the catalyst powders with the POCA powder coating system, ALD and MLD processes were evaluated in the single wafer mode for reference Si (100) substrates (Siltronic Corporation).

### Catalyst Characterization

#### Nitrogen Adsorption and Desorption

The N_2_ physisorption experiments were carried out using a Micromeritics 3Flex 3500 instrument. A catalyst sample (∼300 mg) was placed in the VacPrep degassing station and kept at 150 °C for 12 h under vacuum (10^–2^ mbar). After degassing, the tube was mounted onto the measuring instrument. The catalyst surface area was estimated using the (BET)^51^ equation, and the Barrett–Joyner–Halenda^52^ method was used for total pore volume and average pore diameter determination. Average pore diameter was evaluated from the nitrogen desorption branch.

#### H_2_-TPR and CO-TPD

TPR measurements were performed using a Micromeritics 3Flex 3500 instrument. Before analysis, ∼400 mg of the sample was packed into a quartz U-tube reactor and outgassed in a flow of He at 200 °C. After 2 h, the temperature was decreased to 25 °C, and the temperature program was started until the temperature reached 400 °C with 5 °C/min. The reducing gas was 10% H_2_ in Ar (50 N mL/min). An isothermal hold was maintained for 12 h, and after this, the sample temperature was increased to 900 °C. A thermal conductivity detector (TCD) was used to monitor hydrogen consumption during the temperature program. TPR measurements were performed until assumed complete cobalt reduction at 900 °C. The extent of reduction was estimated by dividing the amount of consumed hydrogen at FT experiment reduction temperature (400 °C) with the amount of hydrogen consumed at assumed full reduction (900 °C). The CO-TPD measurement was conducted using the same apparatus. The sample (100–200 mg) was reduced in H_2_ (60 mL/min) at 400 °C for 12 h. He flush was performed before H_2_ reduction (40 mL/min, 150 °C, 30 min) and after H_2_ reduction (100 mL/min, 120 min). After the He flush and returning the sample temperature to 35 °C, an injection loop was used to pulse a known volume of CO onto the He carrier stream and the CO concentration was monitored with TCD. When the catalyst sample was saturated, a carrier He flush (30 min) removed the residual CO before implementing a temperature ramp up to 950 °C (60 °C/min). CO desorption during the temperature ramp was monitored with TCD and a mass spectrometer (Balzers Omnistar GSD 300 O3). Prior to TC or MS detectors, a cold trap (LN_2_/isopropanol mixture) was used to remove residual water. The shared CO desorption band was determined from deconvolution of the TPD-spectra assuming Gaussian shape of the peaks.

#### Static H_2_ Chemisorption

The H_2_ chemisorption experiments were carried out in a Micromeritics 3Flex 3500 instrument. A catalyst sample (∼200 mg) was first evacuated at 40 °C for 1 h (10^–4^ mbar), followed by reduction with a H_2_ flow of 50 ml/min at 400 °C for 12 h (temperature program 150–400 °C with a 5 °C/min ramp). Subsequently, the samples were cooled down to 100 °C, and H_2_ chemisorption measurements were initiated.

Cobalt metal dispersion was calculated with eq 1,^53^ where χ is the chemisorption H_2_ uptake (μmol/g_cat_), EOR is the extent of reduction from TPR measurement results (see Table 3), and *W* is the mass % of Co in the catalyst. In the calculation, one hydrogen molecule was presumed to interact with two cobalt surface atoms.^53,54^
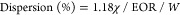
1

The calculated dispersion percentage was used to estimate the cobalt particle size (in nm) in eq 2 by assuming spherical and uniform metal particles with a site density of 14.6 atoms/nm^2^.^55^
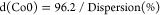
2

#### ICP–MS

A sector field ICP–MS (ThermoScientific Element 2) was used to analyze the concentrations of cobalt in solution. The analysis of Co was performed at medium resolution (*R* ≈ 4500). The Co standards 0.5, 1, 5, 10, and 50 ppb were prepared from multi-elemental reference standard Stock-21 (by Inorganic Ventures). Samples were diluted with a 1/5 ratio into 1% HNO_3_. A control sample was prepared from LPC-1 standard solution (by SPEX). All the measured solutions contained an internal standard—10 ppb of Rhodium—to control the changes in signals. The samples were injected through a SeaSpray nebulizer (0.4 mL/min) and a double-pass spray chamber equipped with a Peltier cooling unit. Washing time between the samples was 3 min with a pump speed of 11 rpm.

### Catalyst Testing

The FT experiments were performed in an automated tubular fixed-bed reactor system (Hastelloy C, 9.1 mm i.d.) at a temperature of 200 °C, a pressure of 20 bar, and a H_2_/CO ratio of 2.0. A detailed equipment description can be found elsewhere.^56^ To minimize temperature gradients over the catalyst bed, ∼0.5 g of the sample (50–150 μm) was diluted with ∼3.0 g of silicon carbide (105 μm). A close to isothermal bed temperature was achieved over the reaction zone (Δ*T* ∼1 °C). An initial reaction temperature runaway was prevented by the slow addition of CO at 190 °C. After the desired inlet gas composition was reached (H_2_ 60 vol %, CO 30 vol %, and N_2_ 10 vol % internal standard), the temperature was increased to 200 °C.

The reaction experiment consisted of five steps, from A to E, as presented in Table 5. In step A, the reaction was started with a dry inlet gas. The initial activity phase was continued until 48 h on stream. In step A, the reaction flows were fixed at 110 N mL/min for all catalysts. Depending on the catalyst activity, the flow rate in step B was adjusted to achieve 15% CO conversion at the reactor outlet. After 24 h of step B, the first co-fed water period (step C) was started with ∼20 mol % added water, and the experiment continued onto step D with increased co-fed water (∼30 mol %). In the last step, step E, co-feeding of water was stopped, and reaction conditions were returned to same as in step B. The co-fed water feeds were selected to represent previous studies:^3−6,11,14^ moderate added water level (20 mol %) with reversible catalyst performance degradation and high added water level (30 mol %) with irreversible catalyst degradation. Corresponding simulated CO conversion levels were 45 and 61% for 20 and 30 mol % added water feeds, respectively.

**Table 5:** Reaction Conditions Summary for Each Step^a^

reaction step	[mol-fraction]	[mol-fraction]	[mol-fraction]	[mol-fraction]	P^0^H_2_O/P^0^H_2_	step duration [h]
A	0.60	0.30	0.10	0	0	48
B	0.60	0.30	0.10	0	0	24
C	0.48	0.24	0.08	0.20	0.42	24
D	0.42	0.21	0.07	0.30	0.71	24
E	0.60	0.30	0.10	0	0	24

a Temperature and pressure were fixed to 200 °C and 20 bar, respectively, in all steps.

FT wax products were collected in a hot trap (150 °C, 20 bar), while water and oil hydrocarbons (C_6_–C_20_) were collected in a Peltier element–cooled liquid–liquid–gas (LLG) separator at 10 °C. Gas compounds (H_2_, N_2_, CO, CO_2_, and C_1_–C_14_ hydrocarbons) continued through the LLG separator onto an on-line gas chromatograph (Shimadzu GC-2030). H_2_, N_2_, CO, CO_2_, and CH_4_ were analyzed with a precolumn (Porapak-Q, 1 mm i.d. × 1.8 m) and an analytical column (Carboxen-1000, 1 mm i.d. × 2.5 m) with a TCD. The heavy hydrocarbons were separated by the precolumn and were backflushed, while light compounds continued to the TCD. The remaining hydrocarbon products from C_1_ to C_14_, as well as C_1_–C_9_*n*-alcohols, were separated and analyzed with a DB-1 capillary column (i.d. 0.25 mm × 60 m × 1 μm) and flame ionization detector.

After sample collection from cold and hot traps, oils and waxes were analyzed offline. Hydrocarbons C_6_–C_20_ and C_1_–C_9_*n*-alcohols were analyzed using a Shimadzu GC-2014 (Rxi-5HT, i.d. 0.32 mm × 30 m × 0.10 μm df). Heavy hydrocarbons (C_10_–C_80+_) were analyzed with a Shimadzu GC-2030 gas chromatograph with an on-column injection port and a CP-SimDist UltiMetal separation column (i.d. 0.53 mm × 10 m × 0.17 μm df, 1 m retention gap). Complete mass balance was calculated by combining online and offline gaseous, oil, and wax analysis results.

## Abbreviations

ALD: atomic layer deposition
FT: Fischer–Tropsch synthesis
ICP–MS: inductively coupled plasma–mass spectrometry
CO-TPD: carbon monoxide proper temperature-programmed desorption
H_2_-TPR: hydrogen temperature-programmed reduction
